# The intronic *BRCA1* c.5407-25T>A variant causing partly skipping of exon 23—a likely pathogenic variant with reduced penetrance?

**DOI:** 10.1038/s41431-020-0612-1

**Published:** 2020-03-20

**Authors:** Hildegunn Høberg-Vetti, Elisabet Ognedal, Adrien Buisson, Tone Bøe Aaman Vamre, Sarah Ariansen, Jacqueline M. Hoover, Geir Egil Eide, Gunnar Houge, Torunn Fiskerstrand, Bjørn Ivar Haukanes, Cathrine Bjorvatn, Per Morten Knappskog

**Affiliations:** 1 Western Norway Familial Cancer Center, Haukeland University Hospital, Bergen, Norway; 2 Department of Medical Genetics, Haukeland University Hospital, Bergen, Norway; 3 Department of Clinical Science, University of Bergen, Bergen, Norway; 4 Hospices Civils de Lyon, Lyon, France; 5 Department of Medical Genetics, Oslo University Hospital, Oslo, Norway; 6 Allegheny Health Network, Pittsburgh, PA USA; 7 Centre for Clinical Research, Haukeland University Hospital, Bergen, Norway; 8 Department of Global Public Health and Primary Care, University of Bergen, Bergen, Norway; 9 Department of Research and Development, Haukeland University Hospital, Bergen, Norway

**Keywords:** Genetics research, Cancer genetics, Medical genetics, Breast cancer, Ovarian cancer

## Abstract

Rare sequence variants in the non-coding part of the *BRCA* genes are often reported as variants of uncertain significance (VUS), which leave patients and doctors in a challenging position. The aim of this study was to determine the pathogenicity of the *BRCA1* c.5407-25T>A variant found in 20 families from Norway, France and United States with suspected hereditary breast and ovarian cancer. This was done by combining clinical and family information with allele frequency data, and assessment of the variant’s effect on mRNA splicing. Mean age at breast (*n* = 12) and ovarian (*n* = 11) cancer diagnosis in female carriers was 49.9 and 60.4 years, respectively. The mean Manchester score in the 20 families was 16.4. The allele frequency of *BRCA1* c.5407-25T>A was 1/64,566 in non-Finnish Europeans (gnomAD database v2.1.1). We found the variant in 1/400 anonymous Norwegian blood donors and 0/784 in-house exomes. Sequencing of patient-derived cDNA from blood, normal breast and ovarian tissue showed that *BRCA1* c.5407-25T>A leads to skipping of exon 23, resulting in frameshift and protein truncation: p.(Gly1803GlnfsTer11). Western blot analysis of transiently expressed BRCA1 proteins in HeLa cells showed a reduced amount of the truncated protein compared with wild type. Noteworthily, we found that a small amount of full-length transcript was also generated from the c.5407-25T>A allele, potentially explaining the intermediate cancer burden in families carrying this variant. In summary, our results show that *BRCA1* c.5407-25T>A leads to partial skipping of exon 23, and could represent a likely pathogenic variant with reduced penetrance.

## Introduction

Since the identification of the *BRCA1* gene in 1994 [1] and the *BRCA2* gene in 1995 [2], clinical testing of these genes has been performed to identify people with high hereditary risk of breast and ovarian cancer [3]. Cancer patients with a pathogenic *BRCA* variant may benefit from targeted therapy with polyadenosine-diphosphate ribose polymerase (PARP) inhibitors, and non-affected carriers may be offered risk-reducing surgery [4, 5]. However, this is not the case if the genetic test reveals a *BRCA* variant of uncertain clinical significance (VUS), which is clinically not actionable according to current guidelines [6]. With increasing use of genetic testing, an increasing number of rare sequence variants are detected. Because of the major clinical impact of distinguishing between a *BRCA* (likely) pathogenic variant and a VUS [6], substantial effort is put into the interpretation of rare variants in diagnostic genetic laboratories every day. Rare variants in non-coding parts of the genes are especially difficult to interpret, and often have limited functional information resulting in a classification of VUS [7].

The *BRCA1* NG_005905.2 (NM_007294.3) c.5407-25T>A variant is located in intron 22 (using traditional numbering 1,2,3,5–24 of the exons). In ClinVar, the variant is registered as VUS by three submitters (https://www.ncbi.nlm.nih.gov/clinvar/variation/371817/). Previously, *BRCA1* c.5407-25T>A has been classified as a VUS in two scientific publications [8, 9], and as likely pathogenic in two other papers, including our recent work on genetic testing results from the DNA-BONus study [10, 11]. Our classification was based on clinical data and cDNA analysis performed in 2006 of the first patient identified with this variant in our diagnostic laboratory. This analysis indicated that *BRCA1* c.5407-25T>A leads to skipping of exon 23 and a subsequently premature stop in exon 24, predicting a non-functional BRCA1 protein (not published).

However, the interpretation of variants that impact splicing is not straightforward. Over the years, there have been several reports of variation in splicing of *BRCA1* [12–15], and the natural occurrence of alternative transcripts needs to be taken into consideration when interpreting variants for their spliceogenic potential [16]. Furthermore, many of these variants may also produce variable amounts of normal transcript in addition to an aberrant transcript, potentially retaining some tumour-suppressor effect [17, 18]. According to suggested guidelines, such variants should be classified as VUS in the absence of clinical evidence of pathogenicity [16, 18, 19].

The aim of this study was to determine the pathogenicity of the *BRCA1* c.5407-25T>A variant by assessing its effect on mRNA splicing combined with allele frequency data and clinical information from families with this variant.

## Materials and methods

### Patient materials and clinical assessment

Families counselled for hereditary breast and ovarian cancer were recruited from Haukeland University Hospital and Oslo University Hospital in Norway, Hospices Civils de Lyon in France and Allegheny Health Network, Pittsburgh, PA in the United States. They were included in the study if the intronic *BRCA1* c.5407-25T>A variant was identified in at least one of the family members before June 1st 2019. All patients signed written informed consent for genetic testing and received genetic counselling. We collected clinical information and family history from the patients’ medical files. To evaluate if the cancer burden was consistent with a potential pathogenic *BRCA* variant running in the family, all families were rated by the Manchester Scoring System [20].

### Control materials

Anonymous blood donors from Haukeland University Hospital were used as controls for DNA and RNA analyses.

### Variant allele frequency in population databases

The variant allele frequencies were retrieved from the gnomAD database (http://gnomad.broadinstitute.org/) as well as from an in-house database containing 784 exome analyses from patients referred to diagnostic testing for different non-cancer disorders. The patients in the in-house database were predominantly Norwegian, and the region of interest had a sequence coverage >20×.

### DNA and RNA analyses

Genomic DNA was extracted from blood using QiaSymphony (Qiagen, Hilden, Germany). RNA was extracted from blood using the PAX-gene kit (Qiagen) and from normal breast and ovary tissues as well as cultured cells using the RNeasy kit (Qiagen). cDNA synthesis was performed using SuperScript (Thermo Fisher Scientific, Waltham, MA, USA) as described by the manufacturer. For RNA splicing analysis, cDNA was PCR-amplified by primers located in exon 17/18 and in the 3′UTR region (BRCA1c5069rnaF2 and BRCA1c5592*158rnaR2, see Supplementary Table S1). The PCR products were analysed by agarose gel electrophoresis and Sanger sequencing. The reference sequence NG_005905.2 (NM_007294.3) and traditional numbering of exons [1–3, 5–24] was used.

The specific expression of *BRCA1* full-length transcript generated from the c.5407-25T>A allele was analysed by assessing the presence of a SNP (c.4837A>G) in *BRCA1* exon 16. For this purpose, the *BRCA1*c4753F primer located upstream of c.4837A>G and the *BRCA1*c5441R primer located in exon 23 (Supplementary Table S1) were used for PCR amplification. The resulting PCR products were analysed by Sanger sequencing and NGS. PCR products produced at different numbers of PCR cycles (30 and 35) were used as template for the Sanger sequencing, and the sequences were analysed using the SeqPilot Software (JSI medical systems, Ettenheim, Germany).

For the NGS analysis, the PCR products generated after 30 cycles of amplification were sequenced using the Illumina Flex library kit and the NextSeq500 sequencer (Illumina, San Diego, CA, USA).

Sequences mapping to the *BRCA1* region were also extracted from data collected after full transcription sequencing of total RNA purified from blood [21], using Illumina TruSeq Stranded Total RNA with Ribo-Zero Globin for library preparation, and sequenced on the HiSeq 4000 Sequencing System (Illumina).

The data described in this study have been submitted to the Leiden Open Variation Database (LOVD v.3.0); https://databases.lovd.nl/shared/variants (DB-ID BRCA1_001122).

### Protein analysis

#### Plasmid constructs

Long-range PCR was performed by Expand^™^ Long Template PCR System (Roche, Mannheim, Germany) using patient-based TA clones containing cDNA corresponding to BRCA1 wild type and p.Gly1803GlnfsTer11 as template, and the primers *BRCA1*kozakwtc1F, *BRCA1*wtc5589* and *BRCA1*c5407-25, as listed in Supplementary Table 1. The final PCR products were TA-cloned into the eukaryotic expression vector pcDNA3.1/V5-His TOPO (Thermo Fisher Scientific), according to the manufacturer’s instructions. The empty vector pcDNA3.1 V5 was used as a negative control. The expected molecular weight including the V5 tag is ~230 kDa for the BRCA1 wild-type protein, and 223 kDa for the predicted truncated protein p.Gly1803GlnfsTer11.

#### Expression of BRCA1 in HeLa cells

HeLa cells (ATCC, Manassas, VA, USA) were cultured in DMEM supplemented with 10% FBS. Transfection was performed using Lipofectamine 2000 (Invitrogen) according to the manufacturer’s instructions. Cells were harvested and lysed in RIPA buffer (for western blot analysis) after 48 h. RNA expression of *BRCA1* (assay ID Hs01556185, Thermo Fisher Scientific) and the two housekeeping genes *GAPDH* (Hs02786624_g1) and *B2M* (Hs00187842_m1) was quantified using TaqMan technology and ABI Prism 7900 HT Fast Real-Time PCR System (Applied Biosystems, Foster City, CA, USA), according to the manufacturer’s protocol.

Controls without template cDNA were included in each assay. Protein samples were separated on NuPAGE 3–8% Tris-acetate protein gels (Thermo Fisher Scientific) and blotted onto nitrocellulose membranes (Thermo Fisher Scientific). The membranes were probed with anti-V5 (Thermo Fisher Scientific) or anti-β-actin (sc-47778, Santa Cruz Biotechnology, Dallas, TX, USA) as loading control in combination with HRP-conjugated second antibody goat anti-mouse (SC 516102, Santa Cruz Biotechnology). Signals were detected using SuperSignal West Pico PLUS Chemiluminescent Substrate (Thermo Fisher Scientific).

## Results

### Patients and clinical assessment

In this study we included 20 apparently unrelated families carrying the *BRCA1* c.5407-25T>A variant, 16 from Norway, three from France and one from USA. In total 66 individuals were proven or obligate carriers of the variant. Among the 49 female carriers, 23 (46.9%) were affected by breast (*n* = 12) or ovarian (*n* = 11) cancer, with mean age of 49.9 years (standard deviation (SD): 9.9, 95% confidence interval (CI): (43.6, 56.2)) at breast cancer diagnosis and 60.4 years (SD 11.3, 95% CI: (52.8, 67.9)) at ovarian cancer diagnosis. Among the 26 unaffected female carriers, seven were 50 years or older without having undergone prophylactic surgery. Key clinical information for the 20 families is summarised in Table 1. The family histories resulted in a mean Manchester score of 16.4 (SD 9.2).

**Table 1:** Characteristics of 20 families from Norway, France and the United States with the *BRCA1* c.5407-25T>A variant.

Family	Index cancer diagnosis	Cancer diagnosis in first-degree relative, if index unaffected	Age at cancer diagnosis of index case^a^	Pathology of index cancer^a^	Manchester score^b^
1	Female breast^c^		45–50	Ductal carcinoma, Er+/Pr+/Her2–, grade 1	4
2		Ovarian	35–40	Not available	14
3		Carcinomatosis, suspected ovarian cancer	55–60	Not available	21
4	Ovarian		45–50	Serous papillary cystadenocarcinoma	30
5	Ovarian		60–65	Serous papillary carcinoma, grade 3	12
6		Breast	30–35	Not available	18
7	Female breast^c^		60–65	Infiltrating ductal carcinoma, Er+/Pr+/Her2–, grade 1	19
8		Breast	65–70	Adenocarcinoma	8
9	Female breast		45–50	Infiltrating ductal carcinoma, triple negative	10
10		Ovarian	60–65	Serous papillary carcinoma, grade 3	12
11	Ovarian		65–70	High-grade serous	15
12	Ovarian		55–60	Granulosa cell tumour	26
13	Female breast		55–60	DCIS, grade 3	12
14	Female breast		50–55	Infiltrating ductal and DCIS, Er+/Pr+/ Her2–	31
15	Ovarian		60–65	High-grade serous	12
16	Male breast		45–50	Invasive ductal adenocarcinoma, grade 2 HR+/Her2–	20
17	Female breast		35–40	Ductal carcinoma, Er+/Pr+/Her2–, grade 3	17
18	Female breast		55–60	Lobular carcinoma, Er+/Pr+/Her2–, grade 2	3
19	Female breast		60–65	Infiltrating carcinoma, triple negative	6
20	Female breast		45–50	Multifocal lobular carcinoma and Er+/Pr+/Her2–, grade 2	37

^a^Diagnosis in first-degree relative, if the index was unaffected.

^b^Combined Manchester score calculated as described in Evans et al. (MSS3) [20].

^c^Previously reported in Høberg-Vetti et al. [10].

### Variant allele frequency

The *BRCA1* c.5407-25T>A variant was identified in 1 out of 400 anonymous blood donors, but was not detected in any of the 784 (non-cancer) patients included in an in-house diagnostic exome database. In the gnomAD database (v2.1.1), the allele frequency of this variant is reported to be 1/141,398 in total and 1/64,566 in the non-Finnish European population (http://gnomad.broadinstitute.org).

### RNA analyses

**Fig. 1:**
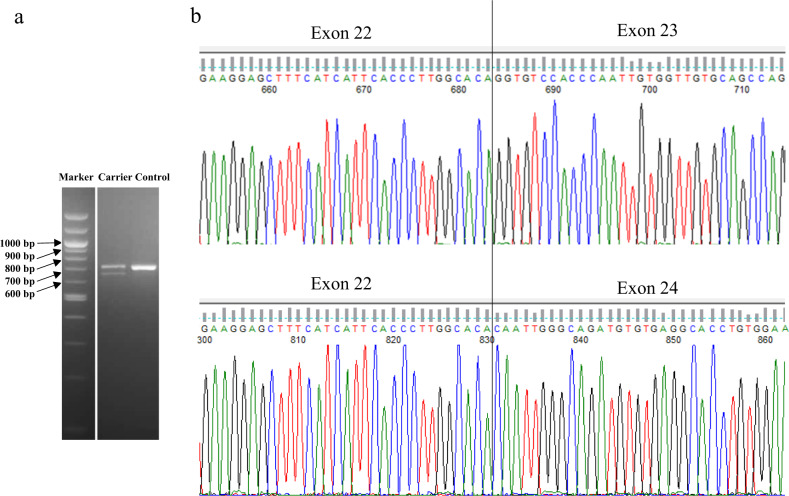
Detection of *BRCA1* transcript lacking exon 23 in *BRCA1* c.5407-25T>A carrier. **a** Agarose gel electrophoresis of PCR products amplified from cDNA using primers located in exon 17/18 and in the 3′UTR showed a single band with the expected length (708 bp) for the control. In the heterozygous carrier of the c.5407-25T>A variant, one additional shorter band of 648 bp was detected. **b** Sanger sequencing of the product from the control confirmed the presence of a wild-type spliced *BRCA1* sequence including exon 23. Sequencing of the two products from the c.5407-25T>A carrier showed both a normal transcript and a transcript lacking exon 23.

cDNA synthesis from blood-derived RNA followed by PCR amplification (of *BRCA1)* and agarose gel electrophoresis showed a single band with the expected wild-type molecular weight in 30 anonymous controls (Fig. 1a). In contrast, two bands were detected in patients heterozygous for the c.5407-25T>A variant (Fig. 1a). DNA sequencing of the PCR product from controls confirmed the presence of a wild-type spliced *BRCA1* sequence containing exon 23. Sequencing of the two products from carriers of the *BRCA1* c.5407-25T>A variant showed both a normal transcript and a transcript lacking exon 23 (r.5407_5467del) (Fig. 1b). This exon skipping (loss of 61 bp) results in a shift in the reading frame and predicts a truncated BRCA1 protein p.(Gly1803GlnfsTer11). In addition, RNA was extracted from normal breast and ovarian tissue from two carriers of the *BRCA1* c.5407-25T>A variant who had undergone risk-reducing surgery. Agarose gel electrophoresis and sequencing of the cDNA- based PCR products showed the presence of both a transcript containing exon 23 and a transcript lacking exon 23, as seen in blood.

Blood-derived RNA from one of the c.5407-25T>A carriers and three normal controls was analysed by full-transcriptome sequencing. Out of ~200 million reads totally in the sample, only a limited number of reads mapped to the *BRCA1* sequence. In the carrier, 4 reads showed a transcript lacking exon 23, and 5 reads showed a transcript containing exon 23. Among the normal controls, there were no transcripts lacking exon 23.

**Fig. 2:**
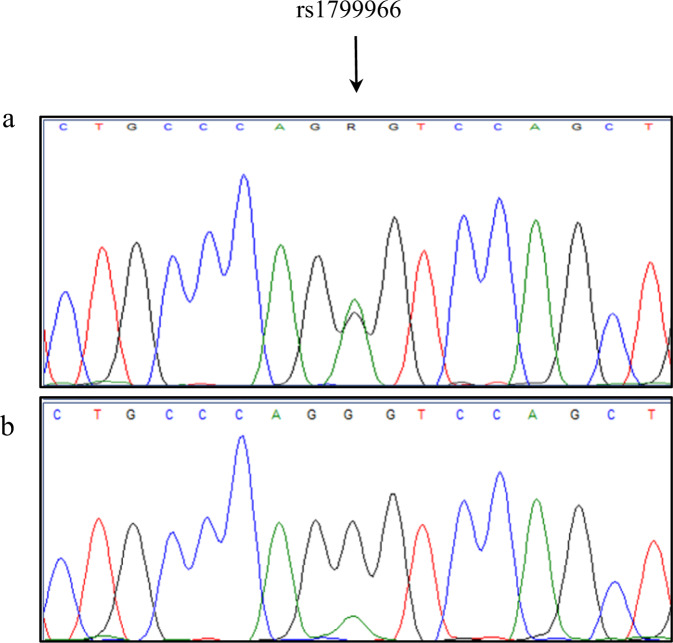
A small amount of correctly spliced transcript including exon 23 is generated from the *BRCA1* c.5407-25T>A allele. **a** *BRCA1* c.5407-25T>A carrier shown to be heterozygous for the SNP rs1799966 (c.4837A>G) in exon 16 by PCR amplification and sequencing of genomic DNA. **b** A region including the SNP rs1799966 was amplified by selective PCR using a reverse primer located within exon 23 and cDNA from the same carrier. Sanger sequencing of only the transcript containing exon 23 showed a small additional peak in the SNP position, indicating that a small amount of correctly spliced transcript including exon 23 is generated from the c.5407-25T>A allele.

Gel electrophoresis of the PCR products indicated that there was some difference in the amount of transcript containing exon 23 and transcript lacking exon 23 (Fig. 1a). To investigate if this could be the result of reduced stability of the transcript lacking exon 23, or caused by only partial mis-splicing of the mRNA transcribed from the variant allele, we studied three individuals heterozygous for a SNP (c.4837A>G) in the transcribed sequence located close to exon 23 in *BRCA1* (Fig. 2). Selective PCR amplification using a reverse primer located within exon 23 followed by Sanger sequencing of only the transcript containing exon 23, showed a small additional peak in the SNP position, demonstrating that a small amount of correctly spliced transcript including exon 23 was generated from the c.5407-25T>A allele. To quantitate the amount of full-length *BRCA1* transcript including exon 23 expressed from the variant allele, the PCR products amplified from RNA (cDNA) were sequenced both by Sanger and NGS technology. NGS-based sequencing of blood-derived RNA from three different carriers showed that the full-length transcript from the variant allele represented 10–13% of the total full-length transcript. Sequencing of RNA isolated from normal breast tissue from one of the carriers showed that full-length transcript from the variant allele represented 20% of the total full-length transcript. Sequencing of blood-derived RNA from a normal control showed that the two alleles were expressed at approximately the same levels (Supplementary Table S2). Comparable results were obtained from Sanger sequencing of PCR products generated after 30 and 35 cycles of amplification (results not shown).

### Protein analysis

Western blot analysis after transient expression in HeLa cells with plasmid encoding wild-type BRCA1 and the truncated protein p.Gly1803GlnfsTer11 is shown in Supplementary Fig. S1. Both proteins revealed bands at expected positions according to molecular weight. After correction for mRNA expression levels, the truncated protein p.Gly1803GlnfsTer11 was less abundant than the wild type. This indicates reduced protein stability for the p.Gly1803GlnfsTer11, as expected for truncated variants.

## Discussion

We have identified the intronic *BRCA1* variant c.5407-25T>A in 20 families recruited from cancer genetics clinics. Based on the results of the current study, we believe this variant is likely pathogenic with possibly reduced penetrance.

Families with *BRCA1* c.5407-25T>A had clinical characteristics resembling other *BRCA*-positive families. The mean age (95% CI) of the onset of breast and ovarian cancer was 49.9 (43.6, 56.2) and 60.4 (52.8, 67.9) years, respectively. This is lower than the corresponding mean age of diagnosis in the general population, which in Norway was 62.1 years for breast cancer and 64.9 years for ovarian cancer in the years 2013–2017, according to the Norwegian Cancer Registry [22]. However, it is higher than reported in a large international prospective study of clinically ascertained carriers of a *BRCA1* pathogenic variant, with median age at diagnosis of breast and ovarian cancer of 44 and 54 years, respectively [23]. On the other hand, a recent large study on unselected women with breast cancer in Sweden found a mean age at diagnosis of 50.3 years in women with a pathogenic *BRCA1* variant [24]. This illustrates that the penetrance of *BRCA1* pathogenic variants may differ, depending on the mode of ascertainment. The carriers of *BRCA1* c.5407-25T>A in our study were ascertained from families undergoing genetic testing and counselling for hereditary breast and ovarian cancer. Following the constant development in genetics, the criteria for selecting patients for *BRCA* genetic testing were more stringent when the first patient with this variant was identified back in 2004, compared with the clinical practice today [4, 5]. As an example, while the Norwegian *BRCA* test criteria in 2004 corresponded to a Manchester score of 16, the 2019 criteria correspond to a Manchester score of 4 [4]. The Manchester scoring system takes into account age-dependent occurrence of cancer in the breasts, ovaries, pancreas and prostate in all family members under the condition of an autosomal-dominant inheritance pattern. In general, a higher Manchester score indicates higher probability of a pathogenic *BRCA* variant being present, e.g. a score above 15 corresponds to 10% probability [20]. We found a mean Manchester score of 16.4 in the 20 families with *BRCA1* c.5407-25T>A. This is noteworthily higher than the mean score of 8.3 in unselected patients with sporadic breast or ovarian cancer and a negative *BRCA* genetic test result (*n* = 462) in our previous DNA-BONus study [10], although the two groups are not directly comparable, given the calculations in the latter group were based on a more limited family history and did not adjust for pathology information. In the same DNA-BONus study, the mean Manchester score was 23.3 in patients where a truncating pathogenic *BRCA* variant was found in previously unrecognised *BRCA* families (*n* = 19). Altogether, these Manchester scores could indicate an intermediate cancer burden in families with *BRCA1* c.5407-25T>A, higher than for average patients with breast and ovarian cancer, but lower than for families with truncating *BRCA* variants.

Despite our finding of the variant in 1 out of 400 anonymous blood donors, the low frequency of 1/64,566 in the normal non-Finnish European population (http://gnomad.broadinstitute.org) indicates that *BRCA1* c.5407-25T>A should not be considered a benign common variant.

In contrast to computer tools that did not predict any effect of this variant on RNA splicing, our results based on RNA extracted from blood, breast and ovarian tissue clearly show that *BRCA1* c.5407-25T>A causes skipping of exon 23. The molecular mechanism for the observed exon 23 skipping is unclear, but the variant may cause a damage to the branch site. However, computer predictions did not show any strong evidence for this mechanism. The exon skipping leads to frameshift and introduction of a premature stop codon in exon 24. In the predicted truncated BRCA1 protein, p.Gly1803GlnfsTer11, the last 61 amino acids are lost, including part of the BRCA1 carboxyl terminal (BRCT) domain that is essential for the tumour-suppressor function of BRCA1 [25]. This is in accordance with our western blot analysis showing a slightly shorter band for the p.Gly1803GlnfsTer11 protein as compared with wild-type BRCA1 (Supplementary Fig. 1). After correction for RNA levels, the p.Gly1803GlnfsTer11 showed lower protein amounts as compared with wild-type protein, indicating reduced protein stability.

Other variants that also lead to skipping of exon 23 have been published as pathogenic, e.g. c.5407-2A>G [11, 26], c.5407-10G>A [27], c.5467+1G>A [28] and c.5434C>G (p.Pro1812Ala) [11, 29]. Although a wide range of alternative transcripts have been described for the *BRCA1* locus [12], there were no signs of aberrant splicing of exon 23 among the 30 normal controls in our study.

Noteworthily, the c.5407-25T>A allele generated a small amount of correctly spliced transcript, including exon 23, in addition to the transcript lacking exon 23 both in blood and breast tissue. According to the three-level classification of splice variants introduced by Houdayer et al. [18], this variant should be considered a class 2S variant (leaky splice site mutation, partial effect). As suggested by Walker et al. [19]. we performed a semi-quantitative calculation of the variant allele contribution to full-length transcript—which showed that 10–13% of the total full-length transcript in blood (*n* = 3) and 20% in breast tissue (*n* = 1) was generated from the c.5407-25T>A allele.

According to previous studies, blood is a relevant source of RNA for analyses of *BRCA1* with respect to breast and ovarian cancer [12, 14, 15]. This is supported by our finding that *BRCA1* c.5407-25T>A leads to skipping of exon 23, both in breast and ovarian tissue, as well as in blood. However, the semi-quantitative analysis indicated a slightly higher proportion of full-length transcript generated from the variant allele in breast tissue (20%) as compared with blood (12%) from the same individual, but we do not know if this represents a true expression difference.

The biological consequence of a small “leakage” of normal transcript from a splice variant is not fully known, but this could lead to synthesis of functional protein and maintenance of some tumour-suppressor effect. Given this background, class 2S splice variants are usually classified as VUS [17–19]. On the other hand, it is reasonable to believe that the decreased amount of normal transcript could lead to a reduced, although not completely absent, tumour-suppressor effect—of potential clinical significance. In line with this, it is possible that the *BRCA1* c.5407-25T>A variant is associated with an increased risk of breast and ovarian cancer, but the magnitude of risk may be lower than for truncating pathogenic *BRCA1* variants. Both the median age at cancer diagnosis and the family histories in our cohort are compatible with a reduced penetrance of *BRCA1* c.5407-25T>A, although we were not able to calculate precise penetrance estimates due to the small number of patients. “Leaky” splice variants have been described to be disease-causing with reduced penetrance for other genes [30–32], and this could also be the case for *BRCA1* and *BRCA2* [17]. However, to our knowledge, the correlation between the ratio of abnormal/normal transcript generated by a variant allele and the magnitude of cancer risk has so far not been investigated systematically for *BRCA* splice variants. The study of de la Hoya et al. indicated that a *BRCA1* allele producing up to 70–80% of transcript encoding tumour-suppressor-deficient protein “may not necessarily confer high-risk of developing cancer” [14]. The classification of “leaky” splice variants is further complicated by the discrepancy of results from different laboratories, most likely due to the use of different techniques and/or experimental conditions. An illustrative example is the *BRCA1* c.5434C>G variant that has been reported to cause complete skipping of exon 23 [18]. Another group showed the same variant to cause partial skipping of exon 23 in about 75% of transcripts [29].

Nevertheless, the *BRCA1* variant c.5434C>G is considered a high-risk pathogenic/likely pathogenic variant by several independent groups [11, 18, 29]. However, most partial splice variants remain VUS [19], leaving patients and doctors in a challenging position. While caution is justified when considering prophylactic surgery in healthy individuals carrying “leaky” *BRCA* splice variants, Gelli et al. claim that such variants in patients with ovarian cancer could serve as indication for treatment with PARP inhibitors [17].

The existence of intermediate-penetrance variants in high-risk genes has been described by the ENIGMA consortium; the missense variant c.5096G>A (p.Arg1699Gln) in *BRCA1* has been demonstrated to be associated with a lower risk of breast and ovarian cancer than truncating variants [33]. Interestingly, we identified *BRCA1* c.5096G>A in one woman with breast cancer and *BRCA1* c.5407-25T>A in two women with breast cancer in our previous DNA-BONus study, where all women with newly diagnosed breast or ovarian cancer were offered *BRCA* genetic testing, regardless of family history or age [10]. One could speculate that a lower threshold for genetic testing potentially could lead to identification of more variants associated with moderate increased cancer risk, not only pathogenic variants in moderate-risk genes, but also variants with reduced penetrance in high-risk genes.

In summary, our results indicate that the intronic *BRCA1* c.5407-25T>A variant causes partly skipping of exon 23, and is a likely pathogenic variant with possible reduced penetrance. Interpretation of splice variants is complex, but should have high priority in diagnostic genetic laboratories. Further studies on an extended number of families with *BRCA1* c.5407-25T>A are warranted to estimate the associated cancer risk, which would be most helpful for the clinical management of patients with this rare intronic variant.

## Supplementary information

Supplementary Information
