# Supplementary material for: The intronic BRCA1 c.5407-25T>A variant causing partly skipping of exon 23—a likely pathogenic variant with reduced penetrance?
Source: Eur J Hum Genet. 2020 Mar 20;28(8):1078–86. doi: 10.1038/s41431-020-0612-1 (PMC7382492; doi:10.1038/s41431-020-0612-1)
Supplement: Supplementary file 1 — Supplementary Information [file 41431_2020_612_MOESM1_ESM.pdf]

Supplementary information for the study of *BRCA1* c.5407-25T>A

Supplementary Table S1

Primer	Sequence 5`-3`
*BRCA1*kozakwtc1F	GAAATGGATTTATCTGCTCTTCGCG
*BRCA1*wtc5589*	GTAGTGGCTGTGGGGGATCTG
*BRCA1*c5407-25	CCACAGGTGCCTCACACATCTG
*BRCA1*c5069rnaF2 (exon17-18)	AAACAGATGCTGAGTTTGTGTG
*BRCA1*c5592*158rnaR2 (UTR)	AGCAGAAAATCTTTAAGGGACC
*BRCA1*c4753F (exon 16)	CCAGAGTCAGCTCGTGTTGG
*BRCA1*c5441R (exon 23)	GCATCTGGCTGCACAACCAC

Primers for PCR amplification.
*Stop codon excluded to allow read trough to C-terminal V5 tag provided by vector.

Supplementary Table S2

Individual	Family	Cancer diagnosis	Tissue	Allele A (%)	Allele G (%)
c.5407-25T>A carrier 1	2	Unaffected	Blood	12%	88%
			Blood	12%	88%
			Breast	20%	80%
c.5407-25T>A carrier 2	7	Breast cancer 60-65 years	Blood	13%	87%
c.5407-25T>A carrier 3	7	Unaffected	Blood	10%	90%
Normal control		Unaffected	Blood	53%	47%

Semi-quantitative analysis of the amount of full-length *BRCA1* transcript including exon 23 expressed from the variant allele: A region including the SNP rs1799966 (c.4837A>G) in exon 16 was amplified by selective PCR using a reverse primer located within exon 23 and cDNA from three carriers of *BRCA1* c.5407-25T>A, and one normal control. The PCR products were sequenced by NGS technology. The table shows the relative contribution of the variant allele and the wild type allele to total full-length *BRCA1* transcript. The samples had a coverage of approximately 100,000x.

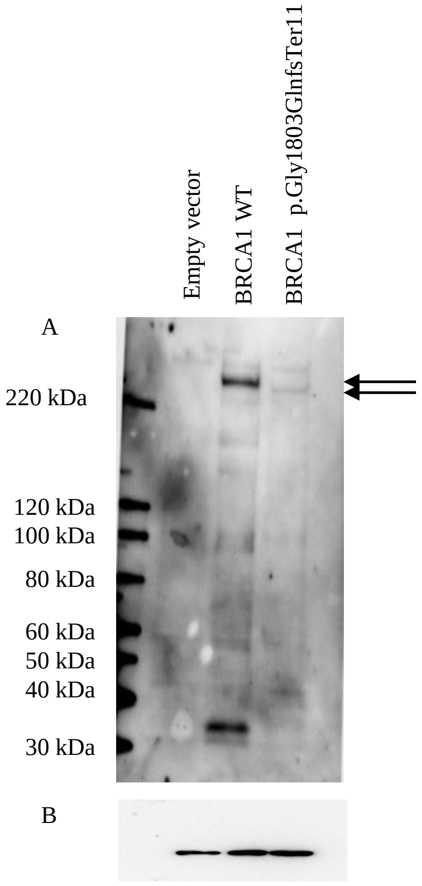

Supplementary Figure S1:
Expression of BRCA1 wild-type and p.Gly1803GlnfsTer11.
HeLa cells were transiently transfected with the empty vector pcDNA3.1 V5 and plasmids encoding BRCA1 wild-type and BRCA1 p.(Gly1803GlnfsTer11) including V5 tag . After 48 hours, cells were lysed and Western blot analysis was performed using A) anti V5, and B) anti-Actin.
